# Identification of novel prognostic and predictive biomarkers in salivary duct carcinoma via comprehensive molecular profiling

**DOI:** 10.1038/s41698-022-00324-1

**Published:** 2022-11-04

**Authors:** Shinji Kohsaka, Yuichiro Tada, Mizuo Ando, Masato Nakaguro, Yukina Shirai, Toshihide Ueno, Shinya Kojima, Hideaki Hirai, Natsuki Saigusa, Satoshi Kano, Kiyoaki Tsukahara, Takafumi Togashi, Hiroyuki Ozawa, Takahito Kondo, Kenji Okami, Hideaki Takahashi, Daisuke Kawakita, Chihiro Fushimi, Takayoshi Suzuki, Akira Shimizu, Isaku Okamoto, Takuro Okada, Yuichiro Sato, Yorihisa Imanishi, Yoshihiro Watanabe, Akihiro Sakai, Koji Ebisumoto, Yukiko Sato, Makoto Urano, Yoshitaka Honma, Keisuke Yamazaki, Yushi Ueki, Toyoyuki Hanazawa, Yuki Saito, Tomotaka Shimura, Toshitaka Nagao, Hiroyuki Mano

**Affiliations:** 1 Division of Cellular Signaling, National Cancer Center Research Institute, 5-1-1 Tsukiji, Chuo-ku, Tokyo, 104-0045 Japan; 2 Department of Head and Neck Oncology and Surgery, International University of Health and Welfare, Mita Hospital, Tokyo, Japan; 3 Department of Otolaryngology-Head & Neck Surgery, Okayama University Graduate School of Medicine, Dentistry and Pharmaceutical Sciences, Okayama, Japan; 4 Department of Pathology and Laboratory Medicine, Nagoya University Hospital, Nagoya, Japan; 5 Department of Anatomic Pathology, Tokyo Medical University, Tokyo, Japan; 6 Department of Otolaryngology Head and Neck Surgery, Faculty of Medicine and Graduate School of Medicine, Hokkaido University, Sapporo, Japan; 7 Department of Otolaryngology Head and Neck Surgery, Tokyo Medical University, Tokyo, Japan; 8 Department of Head and Neck Surgery, Niigata Cancer Center Hospital, Niigata, Japan; 9 Department of Otolaryngology Head and Neck Surgery, Keio University School of Medicine, Tokyo, Japan; 10 Department of Otolaryngology Head and Neck Surgery, Tokyo Medical University Hachioji Medical Center, Tokyo, Japan; 11 Department of Otolaryngology Head and Neck Surgery, Tokai University School of Medicine, Isehara, Japan; 12 Department of Otolaryngology, Head and Neck Surgery, Yokohama City University School of Medicine, Yokohama, Japan; 13 Department of Otolaryngology Head and Neck Surgery, Nagoya City University Graduate School of Medical Sciences, Nagoya, Japan; 14 Department of Otolaryngology and Head and Neck Surgery, School of Life Dentistry at Niigata, The Nippon Dental University, Tokyo, Japan; 15 Division of Pathology, Cancer Institute Hospital, Japanese Foundation for Cancer Research, Tokyo, Japan; 16 Department of Diagnostic Pathology Bantane Hospital Fujita Health University, School of Medicine, Nagoya, Japan; 17 Department of Head & Neck, Esophageal Medical Oncology, National Cancer Center Hospital, Tokyo, Japan; 18 Department of Otolaryngology Head and Neck Surgery, Niigata University Graduate School of Medical and Dental Sciences, Niigata, Japan; 19 Department of Otorhinolaryngology/Head & Neck Surgery, Chiba University Graduate School of Medicine, Chiba, Japan; 20 Department of Otolaryngology - Head and Neck Surgery, Faculty of Medicine, The University of Tokyo, Tokyo, Japan; 21 Department of Otorhinolaryngology, Showa University Fujigaoka Hospital, Yokohama, Japan

**Keywords:** Head and neck cancer, Cancer genomics, Predictive markers, Prognostic markers, Tumour biomarkers

## Abstract

Molecular targets and predictive biomarkers for prognosis in salivary duct carcinoma (SDC) have not been fully identified. We conducted comprehensive molecular profiling to discover novel biomarkers for SDC. A total of 67 SDC samples were examined with DNA sequencing of 464 genes and transcriptome analysis in combination with the clinicopathological characteristics of the individuals. Prognostic biomarkers associated with response to combined androgen blockade (CAB) treatment were explored using mRNA expression data from 27 cases. Oncogenic mutations in receptor tyrosine kinase (RTK) genes or genes in the MAPK pathway were identified in 55 cases (82.1%). Alterations in the phosphatidylinositol 3-kinase (PI3K)/AKT signaling pathway were identified in 38 cases (56.7%). Interestingly, patient prognosis could be predicted using mRNA expression profiles, but not genetic mutation profiles. The risk score generated from the expression data of a four-gene set that includes the *ADAMTS1*, *DSC1*, *RNF39*, and *IGLL5* genes was a significant prognostic marker for overall survival in the cohort (HR = 5.99, 95% confidence interval (CI) = 2.73–13.1, *p* = 7.8 × 10^−6^). Another risk score constructed from the expression of *CD3E* and *LDB3* was a strong prognostic marker for progression-free survival for CAB treatment (*p* = 0.03). Mutations in RTK genes, MAPK pathway genes, and PI3K/AKT pathway genes likely represent key mutations in SDC tumorigenesis. The gene expression profiles identified in this study may be useful for stratifying patients who are good candidates for CAB treatment and may benefit from additional systemic therapies.

## Introduction

Salivary duct carcinoma (SDC) is an aggressive malignancy that resembles high-grade mammary ductal carcinoma^1–3^. SDC was once regarded to account for 1–4% of all salivary gland carcinomas (SGCs)^4–6^, although recent studies have reported a higher incidence than previously recorded^3,7^. SDC usually occurs in major salivary glands such as the parotid gland and is often detected in males greater than 50 years old^8,9^. SDC can arise de novo or as the malignant component of carcinoma ex pleomorphic adenoma (PA)^1,2,10^.

The current treatments for SDC include complete surgical resection of the primary sites followed by adjuvant chemotherapy/radiotherapy^2,3,8,9^. However, recurrence and distant metastasis frequently occur, resulting in a poor prognosis. With conventional therapy, more than 50% of SDC patients die of their disease in 3–5 years, and they are at substantial risk of recurrence even after complete surgical resection. The 5-year overall survival (OS) ranges from 20 to 30%^2,3,6,8,9,11^. Efficacy data are limited for systemic therapy or radiation therapy for individuals with advanced SGC following resection^1,2,8,9^. Reliable prognostic biomarkers are desperately needed to select patients who are at high-risk for recurrence and who may benefit from additional systemic or radiation therapy^1–3^.

Similar to breast carcinoma, conventional immunohistochemistry has identified that SDC often exhibits overexpression of androgen receptor (AR), HER2 (coded by *ERBB2* gene) and epidermal growth factor receptor (EGFR)^8,12–17^. However, SDC usually lacks estrogen receptor- or progesterone receptor-expression which is often observed in breast carcinoma^12,18^. Combined androgen blockade (CAB) with LHRH analog and bicalutamide, a common androgen deprivation therapy (ADT), has a relatively mild toxicity profile and is considered a promising treatment option for patients with unresectable AR–positive SGC. Several clinical trials have demonstrated a favorable response in patients treated with ADT^8,19–22^. In prostate cancer, generation of AR-V7 encoded by a splice variant of *AR* may confer resistance to androgen inhibitor treatment^23^, whereas few specific biomarkers predicting the efficacy of CAB treatment have been identified in SDC^24–28^. For HER2-targeted therapy, a combination of trastuzumab and chemotherapy resulted in impressive response rates in clinical trials^29,30^.

Due to the development of the next-generation sequencing, genetic, transcriptomic, and proteomic profiles have been investigated in SDC^31–36^. Tumor mutational burden is higher than other tumor types of SGC, and the phosphatidylinositol 3-kinase (PI3K)/AKT/mTOR pathway is the most frequently altered pathway^37,38^. Other mutations are frequently found in *TP53*, *HRAS*/*NRAS*, *ERBB2*, *EGFR*, *BRAF* and the CCND1/CDK pathway genes^31,39–43^. Reports have described prognostic factors of the clinical outcomes of SDC^31,39^. The degree of immune cell infiltration in tumor microenvironment is a predictive marker for immune checkpoint inhibitors^34,36^. However, studies have not presented abundant integrative analysis combining comprehensive molecular profiling data with detailed clinical data of SDC patients. Therefore, we performed an integrative analysis combining comprehensive cancer-gene panel along with RNA sequencing (RNA-seq) and analyzed these data in combination with detailed clinical features in a cohort of SDC patients.

## Results

### Patient characteristics

The study cohort comprised 76 SDC patients who underwent surgical resection between 2005 and 2017 at hospitals participating in the Japan SDC consortium. Nine cases were excluded because of poor DNA quality isolated from the specimens. The demographic and clinical data of the remaining 67 individuals, eight patients were female and 59 were male, are summarized in Table 1. The median age was 62.1 years old and was similar between female and male patients (mean, 59.4 vs. 62.6 years; *p* = 0.5). The most common location of the primary tumors was the parotid gland (76.1%) followed by the submandibular gland (20.9%). SDC occurred as a malignant component of carcinoma ex PA in twelve cases (17.9%). More than 50% of the patients had stage IV cancer (62.7 %), and had undergone surgery and chemotherapy/radiotherapy (56.7%). Cancer recurrence was observed in 73.1% of the patients after the initial treatment during the median follow-up time of 40 months. CAB treatment was administered to a total of 27 patients (40.3%).

**Table 1:** Demographic features of the 67 patients with salivary duct carcinoma.

Feature	No. of patients (*N* = 67)
Age in years, median (IQR)	62.1 (55–70)
Range, years	30–94
Age (years), *N* (%)	
<50	15 (22.4)
51–60	11 (16.4)
61–70	25 (37.3)
71–80	14 (20.9)
>80	2 (3)
Sex, *N* (%)	
Female	8 (11.9)
Male	59 (88.1)
Location of primary tumor, *N* (%)	
Parotid gland	51 (76.1)
Submandibular gland	14 (20.9)
Sublingual gland	0 (0)
Minor salivary gland	2 (3)
Tumor arising from pleomorphic adenoma, *N* (%)	
Yes	49 (73.1)
No	12 (17.9)
Not determined	6 (9.0)
T-stage, *N* (%)^a^	
1	12 (17.9)
2	22 (32.8)
3	8 (11.9)
4	25 (37.3)
N-stage, *N* (%)^a^	
0	32 (47.8)
1	4 (6.0)
2	31 (46.3)
3	0 (0)
M-stage, *N* (%)^a^	
0	59 (88.1)
1	8 (11.9)
Overall stage, *N* (%)^a^	
I	7 (10.4)
II	11 (16.4)
III	7 (10.4)
IV	42 (62.7)
Initial treatment, *N* (%)	
Surgery alone	22 (32.8)
Surgery and (chemo-) radiotherapy	38 (56.7)
Chemotherapy and/or radiotherapy	7 (10.4)
Recurrence after initial treatment, *N* (%)	
No persistence or recurrence	18 (26.9)
Recurrence after initial treatment	49 (73.1)
Survival status at last census, *N* (%)	
Alive, no evidence of disease	16 (23.9)
Alive with disease	20 (29.9)
Died of disease	28 (41.8)
Died of other cause	3 (4.5)
Follow-up time in months, median (IQR)	40 (24, 61)

^a^The 8th edition of the UICC (Union for International Cancer Control) staging system, IQR interquartile range.

### Mutational profile of SDC by cancer gene panel sequencing

**Fig. 1:**
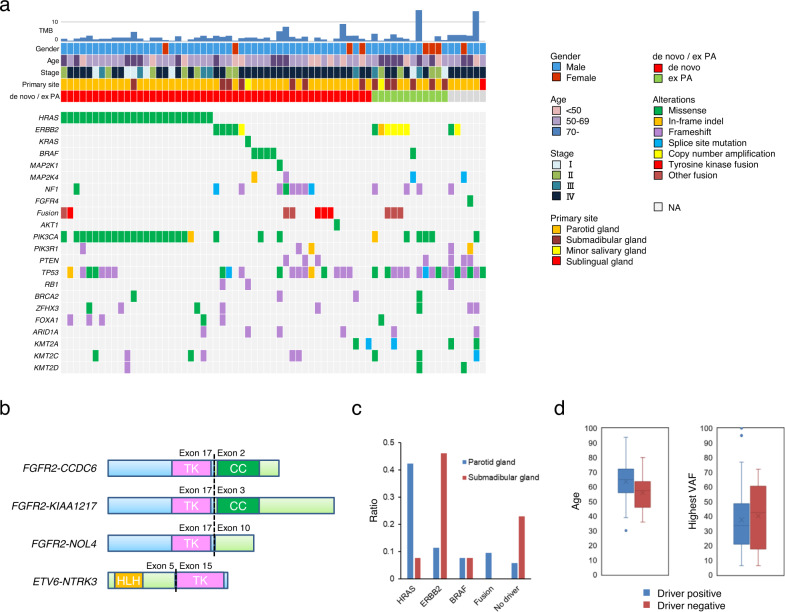
Mutational profile of salivary duct carcinoma. **a** Frequently mutated genes with color coding of their alteration status for each tumor. The gender, age, stage, primary site, tumor origin (de novo or ex pleomorphic adenoma), and tumor mutation burden (TMB) are shown at the top. **b** Schematic diagram depicting tyrosine kinase fusions. *FGFR2*, *RET*, and *NTRK3* fusions identified by RNA-seq are shown with their functional domains. The *FGFR2* gene (NM_000141) was disrupted downstream of exon 17 and was subsequently ligated upstream of either exon 2 of *CCDC6* (NM_005436), exon 3 of *KIAA1217* (NM_019590), or exon 10 of *NOL4* (NM_003787). Exon 15 of *NTRK3* gene (NM_002530) was ligated to exon 5 of *ETV6* (NM_001987). The tyrosine kinase domain (TK) was maintained in all identified fusions. CC, coiled-coil domain; HLH, helix-loop-helix domain. **c** Driver mutations identified in tumors of the parotid and submandibular glands. **d** Comparison of the average age (left) and the highest variant allele frequency (right) of driver mutation-positive and -negative cases. The elements of boxplots are defined as follows: center line, median; lower bound of box, lower quartile; upper bound of box, upper quartile; lower whiskers, minimum value; upper whisker, maximum value.

We used the TOP cancer gene panel^44^ to evaluate somatic single nucleotide variations (SNVs) and insertions/deletions (indels) for 464 genes in the panel, and to determine tumor mutation burden (TMB) and copy number variations. We identified frequent oncogenic mutations within the genes for the receptor tyrosine kinase (RTK)/MAPK signaling pathway including 24 cases with *HRAS* activating mutations, 7 cases with *ERBB2* mutations, 5 cases with *BRAF* mutations, 4 cases with *MAP2K4* mutations, and 13 cases with *NF1* mutations (Fig. 1a). *TP53* was altered in approximately half of the cases. Copy number analysis further identified six cases with *ERBB2* amplification. In total, oncogenic mutations in RTK genes or genes in the MAPK pathway were present in 55 of the cases (82.1%). The list of all mutations found in our cohort is shown as Supplementary Data 1. We included the previous study results by Dalin et al.^31^ to indicate the mutation frequency of individual genes (Supplementary Fig. 1).

In addition, the PI3K pathway was dysregulated in 38 tumors (56.7%) and mutations in *PIK3CA* (29 cases), *PIK3R1* (5 cases), *PTEN* (7 cases) and *AKT1* (1 case) were found in a mutually exclusive manner. *HRAS* mutations were significantly concurrent with *PIK3CA* mutations (83.3%). Other gene mutation identified in multiple cases were *ZFHX3* (8 cases), *FOXA1* (6 cases), *ARID1A* (6 cases), and *KMT2A/C/D* (5, 9, 3 cases, respectively). The average TMB was 2.3 mut/Mb and two cases (2.7%) exhibited hypermutation (>10 mut/Mb).

### Detection of fusion genes in SDC by RNA-seq

RNA-seq identified four cases with tyrosine kinase fusion genes (*FGFR2-CCDC6*, *FGFR2-KIAA1217*, *FGFR2-NOL4*, and *ETV6-NTRK3*) and six cases with other fusions (*CHCHD7-PLAG1*, *FGFR1-PLAG1*, *MAPK14-ZFAND3*, *NFIX-MAST1*, *SLC45A3-ELK4*, *TMCC1-PLXND1*, and *ERBB2-CTTN*) (Fig. 1b and Supplementary Fig. 2).

In total, driver oncogenes, which we defined as oncogenic mutations in receptor tyrosine kinase (RTK) genes or genes in the MAPK pathway, were identified in 55 cases (82.1%).

### mRNA expression analysis in SDC

The expression profile of *ERBB2* established by RNA-seq was compared with the copy number data obtained from the TOP panels, protein expression by immunohistochemistry, and its copy number determined by FISH, and resulted in good concordance among the four assays (Supplementary Fig. 3). Thus, RNA-seq analysis is likely robust for the evaluation of *ERBB2* aberrations. Similarly, the expression of *AR* (*p* = 7 × 10^−4^, low vs. high, Student’s *t* test) and *EGFR* (*p* = 0.02, 3+ vs. the others, Student’s *t* test) both correlated to the results obtained by immunohistochemical analysis. In contrast, *CD274* encoding PD-L1 was more abundantly expressed in driver-negative compared with driver-positive tumors (*p* = 0.01, Student’s *t* test).

### Association of genetic profiles with clinicopathological features

Approximately 40% of the tumors in the parotid gland harbored *HRAS* mutations and 40% of the submandibular tumors were positive for *ERBB2* gene alterations (Fig. 1c). All *HRAS*-mutant tumors (23 cases) were de novo PDC, whereas 50% (6/12) of the tumors from ex PA had *ERBB2* gene alterations.

To evaluate whether the driver mutations were the trunk ones, the variant allele frequency (VAF) of the driver genes was compared with the highest VAF among all mutated genes in each case. For the cases positive for driver mutations of SNVs and indels, the average ratio of driver VAF to the highest VAF was 75.5 (95% CI = 12.6–134), suggesting that the driver mutations are the founding ones in SDC (Supplementary Fig. 4).

There was no significant difference in the highest VAF between the driver-positive and -negative cases (an average of 36.4% vs. 44.8%, respectively, *p* = 0.05, Student’s *t* test) (Fig. 1d). The mean age of the driver-positive cases was slightly older compared with that of driver-negative cases (63.5 years old vs. 55.2 years old, respectively, *p* < 0.05, Student’s *t* test) (Fig. 1d). All clinicopathological features are shown as Supplementary Data 2.

### Transcriptional profiles of SDC

**Fig. 2:**
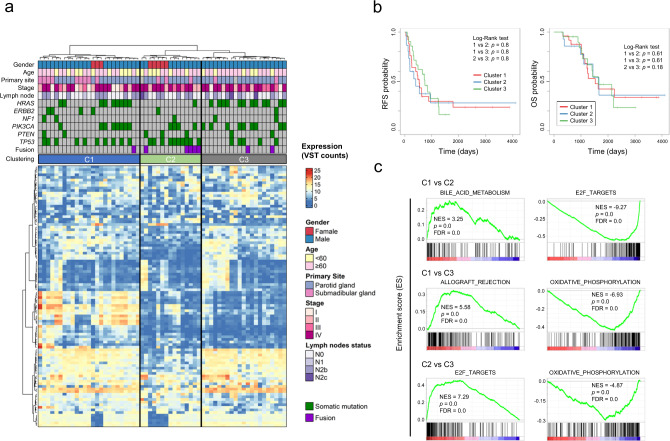
Gene expression profile of salivary duct carcinoma. **a** K-means clustering analysis conducted with RNA-seq data. Clinical information (gender, age, primary site, lymph node involvement, and mutational status) is shown in the upper part. Fisher’s test identified factors associated with either group stratified by k-means clustering. **b** Kaplan–Meier curves of recurrence-free survival in the cohort stratified by k-means clustering as clusters 1–3. **c** GSEA results with the indicated gene sets differentially enriched among clusters defined by k-means clustering. Full GSEA results can be found in Supplementary Data 4.

We conducted a k-means cluster analysis of the tumors based on the gene expression profiles from RNA-seq data, and compared the clusters with clinicopathological features or gene mutational profiles (Fig. 2a). Clustering using the top 100 genes with the most variation of expression divided the cohort into three groups: cluster 1 (the left cluster in Fig. 2a), cluster 2 (middle) and cluster 3 (right) (Supplementary Data 3). Female patients were enriched in cluster 2 (*p* < 0.01, cluster 2 vs. the others, Fisher’s exact test), whereas male patients were enriched in cluster 3 (*p* < 0.05, cluster 3 vs. the others, Fisher’s exact test). There was no significant difference in progression-free survival (PFS) among the clusters (Fig. 2b). The median follow-up time was 40.3 months (range, 2.4–135 months) and the median PFS was 16.4, 11, 23.6 months for cluster 1–3, respectively (95% CI = 16–42.8 months, 6.6–41.1 months and 17.7–30.3 months). The median OS was 40.5, 36.5 and 46.5 months for cluster 1–3, respectively (95% CI = 38.7–64.2 months, 26.2–57.8 months and 37.5–55.6 months).

Gene set enrichment analysis (GSEA) among the three clusters identified 2, 9 and 12 gene sets were upregulated in cluster 1–3, respectively (*q* < 0.01, one cluster vs. the others). Among these gene sets, “E2F_TARGETS” and “HALLMARK_INTERFERON_ RESPONSE” were enriched in cluster 2 while “HALLMARK_OXIDATIVE_PHOSPHORYLATION” was enriched in cluster 3 and “HALLMARK_EPITHELIAL_MESENCHYMAL_TRANSITION” was enriched in cluster 1. The data are bulk transcriptomes and the three clusters might well reflect the microenvironment composition (Fig. 2c and Supplementary Data 4). Expression of salivary gland marker genes including *HTN1/3* and *PRB1/2/3/4* encoding salivary glycoproteins was elevated specifically in cluster 1, suggesting salivary differentiation in the tumors of cluster 1 (Supplementary Data 3).

### Risk assessment for survival after treatment of SDC

Risk assessment was conducted using RNA-seq data from 61 cases. The median follow-up time was 40.5 months (range: 10–135 months) and median OS was 40.5 months (95% confidence interval (CI) = 40.3–54.6 months). For the OS analysis, genes which satisfy the following conditions were selected: gene expression is >0 in more than 80% (49/61) of total samples and sd >1. Univariate Cox proportional hazards regression analysis showed a significant correlation between 9 genes and OS (*p* ≤ 2 × 10^−3^).

**Fig. 3:**
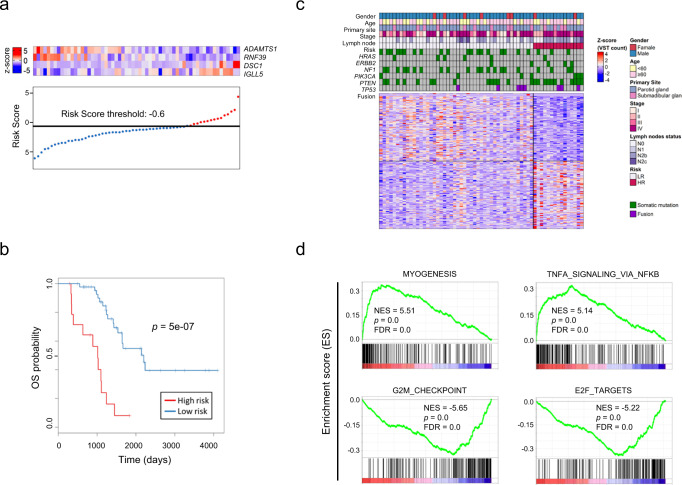
Four-gene prognostic signature of SDC. **a** Four-gene expression and risk score distribution in the cohort by *z*-score. Red and light blue colors indicate higher and lower expression, respectively. Risk scores for all patients are plotted in ascending order and marked as low-risk (blue) or high-risk (red), as divided by the threshold (vertical black line). The risk score threshold was −0.6. **b** Kaplan–Meier curves of overall survival in the cohort stratified by the four-gene prognostic signature for high- and low-risk groups. The log-rank test was used to evaluate *p* values. **c** Heatmap of the top 200 differentially expressed genes between patients at high or low-risk, with red and blue indicating higher and lower expression, respectively. **d** Significantly different gene sets identified by GSEA as differentially overexpressed or underexpressed in high-risk tumors. Supplementary Data 4 presents the full GSEA results. NES normalized enrichment score, FDR false discovery rate.

To generate an OS prognostic signature, these 9 genes were used for forward conditional stepwise regression with multivariable Cox analysis in the cohort. This procedure established a prognostic model with four genes including *ADAMTS1*, *DSC1*, *RNF39*, and *IGLL5*. We constructed a risk score with the regression coefficients from this model and performed manual selection with a suitable threshold at the 75th percentile, which was −0.6 (Fig. 3a). High-risk patients, as defined by the four-gene signature-based risk score, had significantly worse OS (*p* = 5 × 10^−7^, log-rank test) in the cohort (Fig. 3b).

To understand the biology underlying the high-risk tumors, we identified the top 100 significantly overexpressed genes and the top 100 underexpressed genes in the high-risk tumors (Fig. 3c and Supplementary Data 3). There were no differing clinical features between the low- and high-risk groups. However, *TP53* mutations were significantly enriched in the high-risk group as determined by Fisher’s exact test (*p* < 0.01) (Supplementary Fig. 5). The gene sets related to cell proliferation, such as “G2M_CHECKPOINT” and “E2F_TARGETS,” were significantly enriched in the high-risk tumors, whereas gene sets including “MYOGENESIS” and “TNFA_SIGNALING_VIA_NFKB” were enriched in the low-risk group (Fig. 3d and Supplementary Data 4).

Using multivariable Cox analysis, the risk score for OS remained significant (HR = 5.62, *p* = 7.7 × 10^−3^) in the cohort independent of gender, age, primary site, response to first-line treatment, and gene mutations (Table 2). Additional risk factors for OS by univariate analysis included response to first-line treatment (SD; HR = 17.2, *p* = 6.5 × 10^−4^, PD; HR = 7.94, *p* = 2.1 × 10^−4^), *PTEN* mutations (HR = 2.51, *p* = 0.02), *TP53* mutations (HR = 2.74, *p* = 3.0 × 10^−3^), the presence of fusion genes (HR = 2.6, *p* = 0.04), and the N2b/N2c of lymph node status (HR = 2.31, *p* = 0.01); however, none of these factors were significantly different in the multivariate analysis.

**Table 2:** Cox proportional hazard models in salivary duct carcinoma.

			Univariate analysis			Multivariate analysis		
Variables	Categories	No.	Hazard ratio	95% CI	*P* value	Hazard ratio	95% CI	*P* value
Sex	Female	10						
	Male	66	0.82	0.38–2.18	0.82			
Age	<60	28						
	≥60	48	0.99	0.50–1.94	0.97			
Primary site	Parotid gland	56						
	Submadibular gland	17	0.95	0.43–2.08	0.89			
Stage	I	7						
	II	13	0.63	0.11–3.80	0.63			
	III	8	0.87	0.12–6.23	0.89			
	IV	48	2.25	0.54–9.43	0.27			
Results of 1st line treatment	CR	62						
	PR	7	0.94	0.22–3.97	0.94	1.41	0.15–12.9	0.76
	SD	2	17.2	3.31–87.1	6.5E−04	NA	NA	NA
	PD	5	7.94	2.64–23.6	2.1E−04	3.82	0.63–23.3	0.15
Mutations	*ERBB2*	9	0.29	0.07–1.20	0.088			
	*HRAS*	25	0.53	0.25–1.12	0.097			
	*BRAF*	5	0.53	0.13–2.20	0.38			
	*NF1*	9	1.24	0.44–3.53	0.69			
	*PIK3CA*	26	0.58	0.28–1.24	0.16			
	*PTEN*	11	2.51	1.17–5.35	0.02	2.11	0.69–6.43	0.19
	*TP53*	35	2.74	1.41–5.34	3.0E−03	1.16	0.44–3.07	0.77
	Other Genes	15	0.66	0.23–1.87	0.43			
	Fusion	10	2.6	1.04–6.52	0.04	2.1	0.67–6.53	0.2
CAB	F	43						
	T	32	1.62	0.84–3.14	0.15			
Tmab/DTX	F	66						
	T	9	0.33	0.05–2.45	0.28			
Lymph nodes status	N0/N1	41						
	N2b/N2c	35	2.31	1.19–4.48	0.01	1.67	0.69–4.07	0.26
Risk Score (OS)	Low risk	46						
	High risk	15	5.99	2.73–13.1	7.8E−06	5.62	1.58–20.0	7.7E−03

Two-sided likelihood ratio test.

*CAB* combined androgen blockade, *Tmab/DTX* trastuzumab and docetaxel, *OS* overall survival, *CR* complete response, *PR* partial response, *SD* stable disease, *PD* progressive disease, *CI* confidence interval.

### Risk assessment for CAB treatment

Biomarkers related to the response to CAB were derived from the mRNA expression data of 27 cases with CAB treatment. The median follow-up time was 22.2 months (range, 4.2–61.4 months) and the median PFS for CAB treatment was 9.9 months (95% CI = 6.7–13.0 months). Genes which satisfy the following conditions were selected for the analysis: the highest gene expression among the samples is >50. Univariate Cox proportional hazards regression analysis revealed 14 genes that were significantly correlated with PFS (*p* ≤ 2 × 10^−3^).

**Fig. 4:**
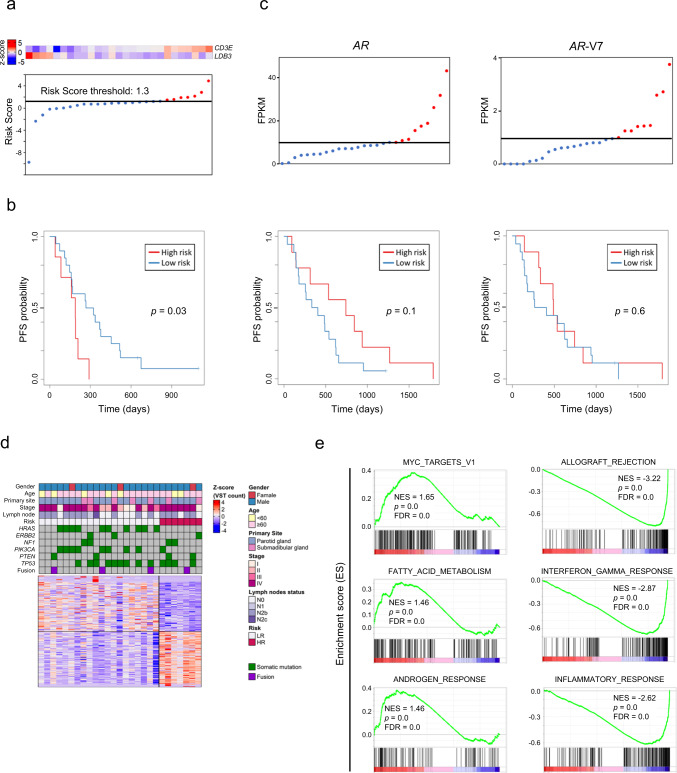
Two-gene predictive signature associated with CAB treatment of SDC. **a** Two-gene expression and risk score distribution in the cohort by *z*-score. Red and light blue indicate higher and lower expression, respectively. Risk scores for all patients are plotted in ascending order and marked as low-risk (blue) or high-risk (red), as divided by the threshold (vertical black line). The risk score threshold was −4.0. **b** Kaplan–Meier curves of recurrence-free survival in the cohort stratified by the two-gene predictive signature in patients at high or low-risk. The log-rank test was used to evaluate *p* values. **c** Kaplan–Meier curves representing progression-free survival (PFS) in the cohort stratified by AR and AR-V7 expression. **d** Heatmap of the top 200 differentially expressed genes between patients at high or low-risk with red and blue indicating higher and lower expression, respectively. **e** Significantly different gene sets identified by GSEA as differentially overexpressed or underexpressed in high-risk tumors. Supplementary Data 4 presents the full GSEA results. NES normalized enrichment score, FDR false discovery rate.

Fourteen genes were used to generate a prognostic signature using a forward conditional stepwise regression with multivariable Cox analysis for the cohort. This procedure established a prognostic model with two genes, *CD3E* and *LDB3*. We constructed a risk score with the regression coefficients from this model and conducted a manual selection of a suitable threshold at the 75th percentile (Fig. 4a). High-risk patients, as defined by the two-gene signature-based risk score, had a significantly worse PFS (*p* = 0.03, log-rank test) compared with the rest of the cohort (Fig. 4b). Neither the *AR-V7* variant expression nor total *AR* expression correlated with patient response to CAB treatment (Fig. 4c and Supplementary Fig. 6).

We repeated the analysis of the differentially expressed genes using the set of patients defined by the two-gene signature to further understand the biology of high-risk tumors (Fig. 4d and Supplementary Data 3). Similar to the OS prognostic signature analyses, gene sets including “MYC_TARGETS_V1,” “FATTY_ACID_METABOLISM” and “ANDROGEN_RESPONSE” were significantly enriched in the high-risk tumors, whereas gene sets including “ALLOGRAFT_REJECTION,” “INTERFERON_GAMMA_RESPONSE” and “INFLAMMATORY_RESPONSE” were enriched in the low-risk group (Fig. 4e and Supplementary Data 4).

Immunohistochemistry of ADAMTS1, DSC1, RNF39, CD3 and LDB3 was performed to evaluate protein expression in tumor cells (Supplementary Fig. 7). The concordance between the FPKM and the protein expression intensity or the ratio of protein expression positive cells was not observed in respective genes (Supplementary Data 5).

## Discussion

This is the first study to identify predictive biomarkers for CAB treatment and prognostic biomarkers of SDC using molecular profiling with next-generation sequencing combined with clinicopathological data. Our analysis identified driver mutations in the RTK/MAPK signaling pathway in 55 out of 67 tumors (82.1%). Given that the driver mutations were truncal, SDC likely depends on these driver genes for growth. There was no difference in the highest VAF of gene mutations between driver mutation-positive and -negative cases; therefore, the latter cases were the true negatives rather than the false negatives caused by low tumor content. Previous comprehensive genomic analysis of 31 SDC cases also revealed driver mutations in ~74% of the cases^31^. Our RNA-seq analysis identified RTK fusions and *ERBB2* overexpression in multiple cases. Furthermore, we precisely depicted the involvement of genes that relate to histone modification, such as *KMT2A/C/D*, and to transcription factors, *ZFHX3* and *FOXA1*, whose expression was identified as a favorable prognosis in our previous study^45^. The TMB of SDC was lower than those found in other solid tumors, which is consistent with a previous study^31^. Further analysis with whole-genome sequencing and long-read sequencing may be useful in deciphering the transformation mechanism of driver-negative SDC.

The utility of RNA-seq was demonstrated by the detection of fusion genes and through expression analyses. The presence of fusion genes was an independent marker for poor OS (HR = 2.6, *p* = 0.04) in this study. Especially, we identified targetable *FGFR1/2* and *NTRK3* fusions in multiple cases. It is promising that there has been recent developments and approval of targeted therapies for cholangiocarcinomas with *FGFR2* fusions and for solid tumors with *NTRK* fusions^46–49^. Evaluation of mRNA expression with RNA-seq was well-correlated with the immunohistochemical staining for HER2, AR, and EGFR. The expression of *CD274* was upregulated in the driver-negative group suggesting that PD-L1 inhibitors may represent a viable treatment option for these patients.

The two-gene signature and the expression of *CD3E* and *LDB3*, but not *AR*, successfully stratified patients by outcome to CAB treatment. *CD3E* encodes CD3-epsilon polypeptide which forms the T-cell receptor-CD3 complex and plays an important role in coupling antigen recognition to several intracellular signal transduction pathways^50^. The immunohistochemistry of CD3 confirmed that the CD3 expression was observed in lymphocytes but tumor cells suggesting CD3E RNA expression probably reflect the tumor immune contexture. The association of infiltrating CD8 + cytotoxic T cells, as well as that of CD3 + T cells, with favorable prognosis has been widely demonstrated in cancers with different histological features and anatomical location, in both primary and metastatic settings, including melanoma, most squamous cell carcinomas, large cell lung cancer and several types of adenocarcinoma^51–54^. The epsilon polypeptide is also involved in T-cell development. *LDB3* encodes a PDZ domain-containing adapter protein in striated muscle to couple protein kinase C-mediated signaling through its LIM domains to the cytoskeleton^55^. What our analysis suggested was that the T-cell infiltration may be a predictive maker for CAB treatment and it was consistent with the previous studies which investigated the immune microenvironment and neoantigen landscape in SDC^34,36^.

Neither *CD3E* and *LDB3* has been reported to be involved in the AR signaling pathway or the metabolism of dihydrotestosterone, a potent agonist of AR. Our GSEA analysis comparing high- and low-risk groups against CAB treatment revealed that the genes involved in androgen pathways are enriched in the low-risk group. This suggests that hormone signal dependency may be another predictive biomarker of the treatment.

We also defined a four-gene set (*ADAMTS1*, *DSC1*, *RNF39*, and *IGLL5*) for predicting aggressive SDC. This gene set may act as a valuable predictive biomarker to stratify patients who may benefit from additional systemic or radiation therapies. These four genes are related to multiple processes, such as inflammation and cell adhesion.

ADAMTS1 is a well-characterized matrix metalloproteinase-related enzyme. However, mammalian cell studies report conflicting roles of ADAMTS1 in cancer development^56^, and different mechanisms have been linked to its protumor/antitumor activities, including the regulation of angiogenesis, lymphangiogenesis, cell proliferation, adhesion, migration, and degradation or interactions with extracellular matrix components. For example, *ADAMTS1* is downregulated in prostate and colorectal cancers by promoter hypermethylation^57^, but is overexpressed in pancreatic cancer^58^, fibrosarcoma^59^, and renal cell carcinoma^60^. Presently, the role of ADAMTS1 in SDC progression remains unclear.

*DSC1* is a desmosomal cadherin that has an important role in cell–cell adhesion^61^. These cadherins include DSC1–3 and four desmogleins. DSC1 was reported to be involved in progression, metastatic potential, and cell adhesion processes in breast cancer^62^. Currently, little is known regarding the involvement of *RNF39* and *IGLL5* in tumorigenesis.

The GSEA analyses identified upregulated expression of genes involved in the G2/M checkpoint, cell cycle-related targets of E2F transcription factors, and epithelial–mesenchymal transition (EMT) in the high-risk group, whereas upregulated genes in the low-risk group were involved in myogenic differentiation. During EMT, epithelial cells lose their cell polarity and cell–cell adhesion, and gain migratory and invasive properties to become mesenchymal stem cells. The enriched gene set related to the cell cycle and EMT may indicate the capability of rapid cell growth, high invasion, and migration of aggressive SDC. Upregulated *ADAMTS1* and *DSC1* expression may have an important role in the survival and invasion of SDC cells, and may explain their association in patients with poor prognosis. Considering that mutations in *TP53* or *PTEN* are also independent markers for poor prognosis, this combination of genetic and epigenetic changes may promote the progression and recurrence of SDC.

The discordance between protein expression and RNA-seq data is because the sensitivity of RNA-seq to quantify RNA expression might be higher compared with immunohistochemistry to quantify protein expression. In fact, the protein expression levels of DSC1, LDB3 and RNF39 in most samples were too low to be detected by immunohistochemistry while ADAMTS1 expression was moderately positive in all samples. The CD3 protein expression was observed in lymphocytes but tumor cells suggesting CD3E RNA expression probably reflect the tumor immune contexture. Although the concordance was not observed in these genes, we are confident that our RNA-seq is suitable for the evaluation of RNA expression as good correlations were observed in HER2, AR and EGFR expression (Supplementary Fig. 3). RNA-seq may be preferable method to evaluate expression of genes whose protein level are low or when the immunohistochemical analysis is not well optimized.

A major limitation of this study is that this a retrospective study without area under the curve assessment supporting the specificity of the biomarker signature. Therefore, prospective studies of independent cohorts should be conducted to validate the proof of concept. Another limitation is about TOP panel that it can’t detect all the mutations exiting in the cancer and doesn’t necessarily elucidate all tumor features. Therefore, comprehensive assays such as whole-genome sequencing and genome wide methylation analysis are needed to identify additional novel biomarkers.

In conclusion, our discoveries may have the potential for application in a clinical setting. Expression profiling may directly predict the efficacy of CAB treatment and the prognosis of SDC. Our genomic and transcriptomic analyses highlight the importance of precise tumor profiling to provide increased treatment options for SDC patients.

## Methods

### Study design and patient specimens

The study cohort consisted of 76 SDC patients who underwent surgical resection between October 2005 and September 2017 at hospitals in the Japan SDC consortium, including the International University of Health and Welfare, Mita Hospital, Tokyo Medical University Hospital, Tokyo Medical University Hachioji Medical Center, Hokkaido University, Niigata Cancer Center Hospital, Keio University, and Tokai University. Nine patients were excluded because of poor quality DNA and the analysis was conducted in the remaining 67 patients. Several cases were previously reported by Otsuka et al.^9^, Takase et al.^16^, Masubuchi et al.^17^, Shimura et al.^41^, Fushimi et al.^20^, Takahashi et al.^29^, and Okada et al.^63^. A board-certified pathologist (TN) specializing in salivary gland tumors reviewed the SDC histological features based on the criteria of the current World Health Organization classification. Fresh frozen specimens or formalin-fixed paraffin-embedded (FFPE) of surgically resected tumors were obtained from all patients. Approval for this study was obtained from the Ethics Committee of National Cancer Center (No. 2019-271), International University of Health and Welfare, Mita Hospital (No. 5-19-6), Tokyo Medical University (No. SH2563), Faculty of Medicine and Graduate School of Medicine, Hokkaido University (No. 017-0487), Keio University School of Medicine (No. 20120083), School of Medicine, Tokai University (No. 20R-204), Tokyo Medical University Hachioji Medical Center (No. SH2563) and Niigata Cancer Center Hospital (No. 2021-300). All subjects provided written informed consent, except for those who could not be reached because of loss of follow-up or death at registration. In these cases, the Institutional Review Board at each participating institution granted permission for the existing tissue samples to be used for research purposes. No samples from the patients who had opted out of participation were used in this study.

### Immunohistochemistry and fluorescence in-situ hybridization (FISH)

Immunohistochemistry and FISH were conducted as previously described^16,41^. Briefly, FFPE tumor tissues were cut into 3 μm-thick sections, and a polymer-based detection system with heat-mediated antigen retrieval was used with primary antibodies against HER2 (Polyclonal, cat# A0485, Agilent Technologies, Santa Clara, CA) with 1:400 dilution, AR (clone AR441, cat# PM109AA, BIOCARE Medical LLC, CA), EGFR (clone 31G7, cat# 423701, NICHIREI BIOSCIENCES INC, Tokyo, Japan) in undiluted form, PD-L1 (clone 22C3, cat# M3653, Agilent Technologies) with 1:50 dilution, ADAMTS1 (cat# 12749-1-AP, Proteintech, Rosemont, IL) with 1:50 dilution, DSC1(clone A-4, cat# sc-398590, Santa Cruz Biotechnology, Dallas, TX) with 1:100 dilution, RNF39 (cat# HPA047115, Atlas Antibodies, Bromma, Sweden) with 1:200 dilution, CD3 (clone SP-7, cat# 413601, Nichirei Biosciences, Tokyo, Japan) with 1:100 dilution, and LDB3 (cat# 11004-1-AP, Proteintech) with 1:100 dilution. Diaminobenzidine was used to detect antigen-antibody reactions. Appropriate positive and negative controls were applied for all conditions. *ERBB2* amplification was evaluated using PathVysion HER-2 DNA Probe Kit (cat# 02J01-031, Abbott Molecular, Des Plaines, IL). For the PD-L1 IHC 22C3 pharmDx assay, the slides were stained using a Dako Autostainer Link 48 platform with an automated staining protocol and the pathologists A. U. and T. H. used a light microscope to score the percentage of positive tumor cells in each sample.

### Evaluation of HER2 status

HER2 positivity was defined as either immunohistochemically 3+ or *ERBB2* amplification according to the American Society of Clinical Oncology/College of American Pathologists guidelines for breast cancer^64^. In Her2 Immunohistochemistry, 3+ stain was defined as a circumferential membrane staining that was complete and intense and exhibited >10% frequency of tumor cells. For *ERBB2* copy number prediction with FISH analysis, 100 non-overlapping, intact interphase tumor nuclei stained with 4′,6-diamidino-2-phenylindole (DAPI) were evaluated. The *ERBB2* gene (red signal) and *CEP17* (green signal) copy numbers in each nucleus were assessed. *ERBB2* amplification is annotated when the average *ERBB2*/*CEP17* ratio was ≥2.0 in all nuclei or when the *ERBB2* signals formed a tight gene cluster.

### Assessment of immunohistochemistry of AR, EGFR, ADAMTS1, DSC1, RNF39, CD3 and LDB3

A case was considered positive for AR when ≥20% of tumor cell nuclei exhibited strong staining. The percentage of EGFR immunostaining cells was scored from 0 to 3+ as follows: 0, 0%; 1+, 1–10%; 2+, 11–30%; and 3+, >30%. Score 3+ was considered positive for EGFR. For ADAMTS1, DSC1, RNF39, CD3 and LDB3, staining intensity and ratio of positive cells were assessed. The staining intensity was scored from 0 to 3+ as follows: 0, negative; 1+, mildly positive; 2+, moderately positive; 3+, strongly positive.

### DNA sequencing with the TOP cancer gene panel for mutation call and copy number analysis

Fresh frozen or FFPE SDC specimens were analyzed with TOP panel version 3^44^. This evaluates nucleotide variants and insertions/deletions for 464 genes to calculate TMB and to infer copy number variation. Genomic DNA was extracted from FFPE samples using the GeneRead DNA FFPE Kit (Qiagen, Hilden, Germany) and 500 ng of each DNA sample was used for target fragment enrichment with an Agilent Kit (v6) (Agilent Technologies). Next-generation sequencing was conducted with a HiSeq2500 (Illumina) using a paired-end option and sequencing reads were independently aligned to the human reference genome (hg38) using BWA^65^, Bowtie2 (http://bowtie-bio.sourceforge.net/bowtie2/index.shtml), and NovoAlign (http://www.novocraft.com/products/novoalign/). Somatic mutations were called using MuTect (http://www.broadinstitute.org/cancer/cga/mutect), SomaticIndelDetector (http://www.broadinstitute.org/cancer/cga/node/87), and VarScan (http://varscan.sourceforge.net). The exclusion criteria for mutation analysis were (i) the read depth was <20 or the VAF was <0.1; (ii) mutations were supported by only one strand of the genome; or (iii) they were SNP in either the 1000 Genomes Project dataset (http://www.internationalgenome.org/) or our in-house database. Gene mutations were annotated by SnpEff (http://snpeff.sourceforge.net). Copy number was evaluated using our in-house pipeline to calculate the logR ratio (LRR) as follows: (i) used selected homozygous (VAF, ≤ 0.05 or ≥0.95) or a heterozygous (VAF, 0.4–0.6) SNP in the 1000 Genomes Project database; (ii) normal and tumor read depths at the selected SNP position were counted and adjusted based on GþC percentage around 100 bp from the position^66^; (iii) calculated the LRR = log_2_ (*t*_*i*_/*n*_*i*_), where *n*_*i*_ and *t*_*i*_ are the normal and tumor-adjusted depths at the SNP position; and (iv) each representative LRR was determined by the median of a moving window (1 Mb) around the SNP position. The values of the LRR of the copy number of the major allele and the minor allele were determined for every region of the entire genome. The *p* values for the gain or loss of respective genomic regions were determined from the LRRs with a permutation test (100,000 iterations) according to the algorithm used in GISTIC^67^, and *q* values were calculated using the R package *q* value (http://github.com/jdstorey/qvalue).

### Transcriptome sequencing, expression analysis, and detection of fusion genes and exon skipping

Total RNA was extracted from fresh, frozen, or FFPE samples using RNA-Bee (Tel-Test Inc., Gainesville, FL) and treated with DNase I (Thermo Fisher Scientific, Waltham, MA). The RNA-seq library was prepared with a TruSeq RNA Access Library Prep Kit (Illumina, San Diego, CA). Sequencing was conducted from both ends of each cluster using a HiSeq 2500 (Illumina). RNA-seq was aligned to hg19 using TopHat (v2.0.9; https://ccb.jhu.edu/software/tophat/index.shtml). Gene expression was quantified using Cufflinks (v2.1.1; http://cole-trapnell-lab.github.io/cufflinks) and gene fusions were explored using the deFuse pipeline (https://bitbucket.org/dranew/defuse). The fusion genes which matched the following criteria were selected: fusion genes reported in COSMIC or fusions genes of kinase in the RTK/MAPK signaling pathway and the fusion split reads were >10. Exon skipping was analyzed using an in-house pipeline developed in the previous study^68^ according to the following steps: (i) aligned RNA-seq reads to hg38 and the NCBI reference sequence (RefSeq) using Burrows–Wheeler Aligner and Bowtie2; (ii) detected skipped exons in the mapped RefSeq data; (iii) created virtual transcriptome sequences dynamically; (iv) aligned RNA-seq reads to candidate transcriptome sequences; and (v) identified exon skipping candidates based on reads with a breakpoint.

### Sanger sequencing

For capillary sequencing with a 3130xl Genetic Analyzer (Thermo Fisher Scientific), PCR products prepared from 10 ng of template cDNA were used to amplify *FGFR2-CCDC6*, *FGFR2-KIAA1217*, *FGFR2-NOL4*, *ETV6-NTRK3*, *MAPK14-ZFAND3*, *NFIX-MAST1*, *SLC45A3-ELK4*, *TMCC1-PLXND1*, and *ERBB2-CTTN* by GoTaq G2 Hot Start Master Mix Green (Promega, Madison, WI), in accordance with the manufacturer’s instructions with the following primers: 5′-GCAGTTGGTAGAAGACTTGGATCG-3′ and 5′-GCAAGGGTTTCTTTCTCCTTCTGC-3′ for *FGFR2-CCDC6*, 5′-GCAGTTGGTAGAAGACTTGGATCG-3′ and 5′-GACGTCTCTGATAAATGCTCGACC-3′ for *FGFR2-KIAA1217*, 5′-GCAGTTGGTAGAAGACTTGGATCG-3′ and 5′-ACAGCTGATGTTGAGTAAGTGGCC-3′ for *FGFR2-NOL4*, 5′-GAACCACATCATGGTCTCTGTCTC-3′ and 5′-CAGGAAGACCTTTCCAAAGGCTC-3′ for *ETV6-NTRK3*, 5′-GAACCTACAGAGAACTGCGGTTAC-3′ and 5′-TACTTGGAGCGGAATCATCGTCTG-3′ for *MAPK14-ZFAND3*, 5′-GCCACATCACATTGGAGTCACAATC-3′ and 5′-CAGCGCTCCAATATTCTTCAGCAG-3′ for *NFIX-MAST1*, 5′-TTCACCTTCTCAGCCCTGCAGATC-3′ and 5′-TTCTGCAGGAGCTGAAGAAGGAAC-3′ for *SLC45A3-ELK4*, 5′-TGTTCCATCCACAGAGTCTGTGTG-3′ and 5′-ACTCCTGTGTTTCCAACCAGTCTC-3′ for *TMCC1-PLXND1*, 5′-ATAACACCCACCTCTGCTTCGTG-3′ and 5′-CATACTTCCCGCCGAATCCTTTG-3′ for *ERBB2-CTTN*.

### Signature generation and statistical analysis

The duration of OS for the risk assessment of survival was defined as the period between the beginning of treatment and the date of death from any cause or the last follow-up. For the OS analysis, genes which satisfy the following conditions were selected: gene expression is >0 in more than 80% (49/61) of total samples and sd >1. After this initial filtering, the *p* value was calculated and nine genes were *p* ≤ 0.002 and further analyzed for forward conditional stepwise regression with multivariable Cox analysis in the cohort. The duration of PFS for the risk assessment of CAB was defined as the period from the day of initiation of CAB treatment to the day of progressive disease or death. For the PFS analysis of CAB treatment, genes which satisfy the following conditions were selected: the highest gene expression among the samples is >50. After this initial filtering, the *p* value was calculated and 14 genes were *p* ≤ 0.002 and further used to construct a predictive model. Candidate genes were fitted in a stepwise multivariate Cox regression analysis to evaluate their relative contribution to survival prediction in the cohort. Genes that correlated with survival were included in the prognostic signature. According to the estimated regression coefficients in multivariate Cox regression analysis, a prognostic risk scores for predicting OS and PFS were calculated as similar to previous studies^66^. All statistical analyses were conducted using R software (version 3.5.1; https://www.r-project.org/) and relevant packages. Survival analysis and Cox regression analyses were conducted using the “survival” (v2.44.1.1) package. OS and PFS were analyzed using the Kaplan–Meier method and curve differences were evaluated using the log-rank test according to either the risk score or the driver mutation subtypes. GSEA was conducted using Java GSEA software (http://software.broadinstitute.org/gsea/index.jsp) (v2.2.4).

### Reporting summary

Further information on research design is available in the Nature Research Reporting Summary linked to this article.

## Supplementary information

REPORTING SUMMARY

Supplementary Figures 1-7

Supplementary Data 1

Supplementary Data 2

Supplementary Data 3

Supplementary Data 4

Supplementary Data 5

## Data Availability

We have deposited the raw sequencing data of all new and unpublished DNA and RNA sequencing data presented in this paper under accession number hum0094 in the Japanese Genotype-Phenotype Archive which is hosted by the DNA Data Bank of Japan, under accession number JGAS000534.

## Code availability

No unreported custom computer code or algorithm was used to generate the results of this paper. The references of all source codes are included within Materials and Methods.
