# Supplementary material for: Identification of novel prognostic and predictive biomarkers in salivary duct carcinoma via comprehensive molecular profiling
Source: NPJ Precis Oncol. 2022 Nov 4;6:82. doi: 10.1038/s41698-022-00324-1 (PMC9636405; doi:10.1038/s41698-022-00324-1)
Supplement: Supplementary file 1 — REPORTING SUMMARY [file 41698_2022_324_MOESM1_ESM.pdf]

nature portfolio

Corresponding author(s): Shinji Kohsaka

Last updated by author(s): Oct 4, 2022

Reporting Summary

Nature Portfolio wishes to improve the reproducibility of the work that we publish. This form provides structure for consistency and transparency in reporting. For further information on Nature Portfolio policies, see our Editorial Policies and the Editorial Policy Checklist.

Statistics

For all statistical analyses, confirm that the following items are present in the figure legend, table legend, main text, or Methods section.

n/a	Confirmed	
☐	☒	The exact sample size (n) for each experimental group/condition, given as a discrete number and unit of measurement
☐	☒	A statement on whether measurements were taken from distinct samples or whether the same sample was measured repeatedly
☐	☒	The statistical test(s) used AND whether they are one- or two-sided<br>*Only common tests should be described solely by name; describe more complex techniques in the Methods section.*
☐	☒	A description of all covariates tested
☐	☒	A description of any assumptions or corrections, such as tests of normality and adjustment for multiple comparisons
☐	☒	A full description of the statistical parameters including central tendency (e.g. means) or other basic estimates (e.g. regression coefficient) AND variation (e.g. standard deviation) or associated estimates of uncertainty (e.g. confidence intervals)
☐	☒	For null hypothesis testing, the test statistic (e.g. F, t, r) with confidence intervals, effect sizes, degrees of freedom and P value noted<br>*Give P values as exact values whenever suitable.*
☒	☐	For Bayesian analysis, information on the choice of priors and Markov chain Monte Carlo settings
☐	☒	For hierarchical and complex designs, identification of the appropriate level for tests and full reporting of outcomes
☒	☐	Estimates of effect sizes (e.g. Cohen's d, Pearson's r), indicating how they were calculated

Our web collection on statistics for biologists contains articles on many of the points above.

Software and code

Policy information about availability of computer code

Data collection	No unreported custom computer code or algorithm was used to generate the results of this manuscript.
Data analysis	N/A

For manuscripts utilizing custom algorithms or software that are central to the research but not yet described in published literature, software must be made available to editors and reviewers. We strongly encourage code deposition in a community repository (e.g. GitHub). See the Nature Portfolio guidelines for submitting code & software for further information.

Data

Policy information about availability of data

All manuscripts must include a data availability statement. This statement should provide the following information, where applicable:

- Accession codes, unique identifiers, or web links for publicly available datasets
- A description of any restrictions on data availability
- For clinical datasets or third party data, please ensure that the statement adheres to our policy

We have deposited the raw sequencing data in the Japanese Genotype-Phenotype Archive (http://trace.ddbj.nig.ac.jp/jga), which is hosted by the DNA Data Bank of Japan.

Field-specific reporting

Please select the one below that is the best fit for your research. If you are not sure, read the appropriate sections before making your selection.

☒ Life sciences ☐ Behavioural & social sciences ☐ Ecological, evolutionary & environmental sciences

For a reference copy of the document with all sections, see nature.com/documents/nr-reporting-summary-flat.pdf

Life sciences study design

All studies must disclose on these points even when the disclosure is negative.

Sample size	Sample size was described in the manuscript.
Data exclusions	Data exclusions were properly described in the manuscript.
Replication	For next-generation sequencing, no replicate was performed.
Randomization	Randomization was not performed for this study.
Blinding	Blinding in this study was not performed. Data analysis were performed by the computational pipeline.

Reporting for specific materials, systems and methods

We require information from authors about some types of materials, experimental systems and methods used in many studies. Here, indicate whether each material, system or method listed is relevant to your study. If you are not sure if a list item applies to your research, read the appropriate section before selecting a response.

Materials & experimental systems

n/a	Involved in the study	
☐	☒	Antibodies
☒	☐	Eukaryotic cell lines
☒	☐	Palaeontology and archaeology
☒	☐	Animals and other organisms
☐	☒	Human research participants
☒	☐	Clinical data
☒	☐	Dual use research of concern

Methods

n/a	Involved in the study	
☒	☐	ChIP-seq
☒	☐	Flow cytometry
☒	☐	MRI-based neuroimaging

Antibodies

Antibodies used	Primary antibodies against HER2 (Polyclonal, cat# A0485, Agilent Technologies, Santa Clara, CA) with 1:400 dilution, AR (clone AR441, cat# PM109AA, BIOCARE Medical LLC, CA), EGFR (clone 31G7, cat# 423701, NICHIREI BIOSCIENCES INC, Tokyo, Japan) in undiluted form, PD-L1 (clone 22C3, cat# M3653, Agilent Technologies) with 1:50 dilution, ADAMTS1 (cat# 12749-1-AP, Proteintech, Rosemont, IL) with 1:50 dilution, DSC1(clone A-4, cat# sc-398590, Santa Cruz Biotechnology, Dallas, TX) with 1:100 dilution, RNF39 (cat# HPA047115, Atlas Antibodies, Bromma, Sweden) with 1:200 dilution, CD3 (clone SP-7, cat# 413601, Nichirei Biosciences, Tokyo, Japan) with 1:100 dilution, and LDB3 (cat# 11004-1-AP, Proteintech) with 1:100 dilutionwere used for immunohistochemistry.
Validation	The antibody used in immunohistochemistry was obtained from commercial sources. Please see manufacturer's link for validation of antibody.

Human research participants

Policy information about studies involving human research participants

Population characteristics	The study cohort consisted of 76 SDC patients who underwent surgical resection between October 2005 and September 2017 at hospitals in the Japan SDC consortium, including the International University of Health and Welfare, Mita Hospital, Tokyo Medical University Hospital, Tokyo Medical University Hachioji Medical Center, Hokkaido University, Niigata Cancer Center Hospital, Keio University, and Tokai University. Nine patients were excluded because of poor quality DNA and the analysis was conducted in the remaining 67 patients.
Recruitment	The study cohort consisted of 76 SDC patients who underwent surgical resection between October 2005 and September 2017 at hospitals in the Japan SDC consortium.
Ethics oversight	Approval for this study was obtained from the Ethics Committee of National Cancer Center (No. 2019-271), International

Ethics oversight	University of Health and Welfare, Mita Hospital (No. 5-19-6), Tokyo Medical University (No. SH2563), Faculty of Medicine and Graduate School of Medicine, Hokkaido University (No. 017-0487), Keio University School of Medicine (No. 20120083), School of Medicine, Tokai University (No. 20R-204), Tokyo Medical University Hachioji Medical Center (No. SH2563) and Niigata Cancer Center Hospital (No. 2021-300).

Note that full information on the approval of the study protocol must also be provided in the manuscript.
