# Supplementary material for: Identification of novel prognostic and predictive biomarkers in salivary duct carcinoma via comprehensive molecular profiling
Source: NPJ Precis Oncol. 2022 Nov 4;6:82. doi: 10.1038/s41698-022-00324-1 (PMC9636405; doi:10.1038/s41698-022-00324-1)
Supplement: Supplementary file 2 — Supplementary Figures 1-7 [file 41698_2022_324_MOESM2_ESM.docx]

**Supplementary Figures**

**
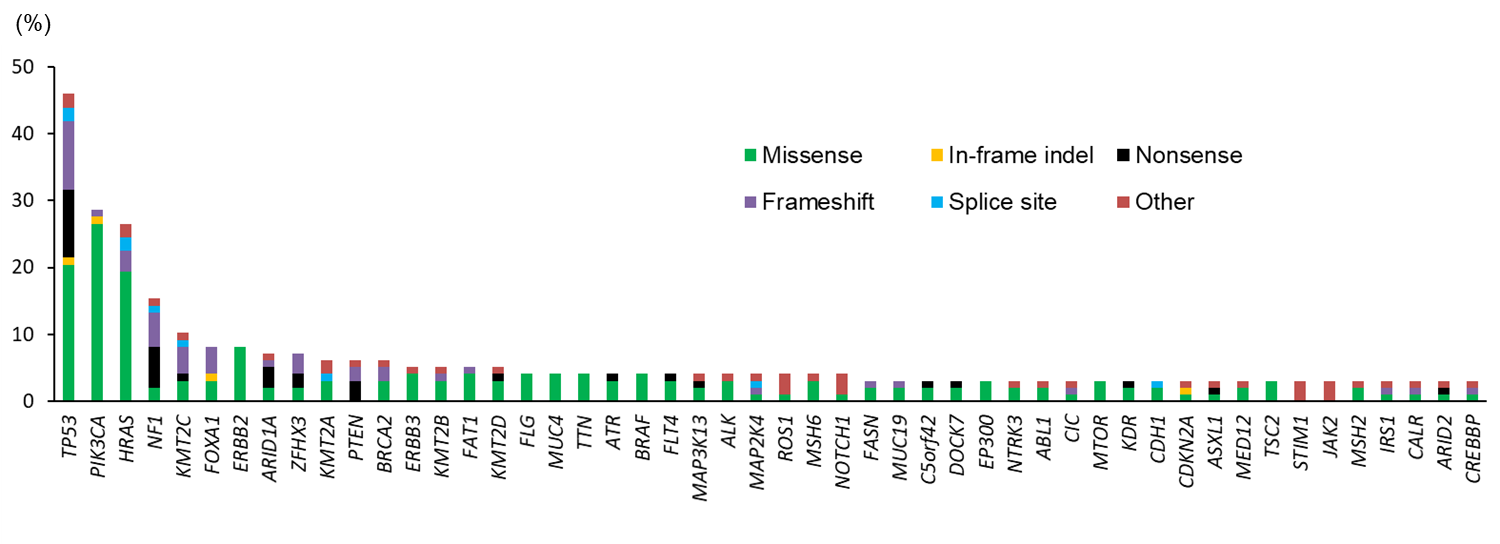
**

**Supplementary Figure 1. The mutation frequency of individual genes**

The mutation data of this cohort and the previous cohort of Dalin et al. were combined to indicate the mutation frequency of individual genes.

**
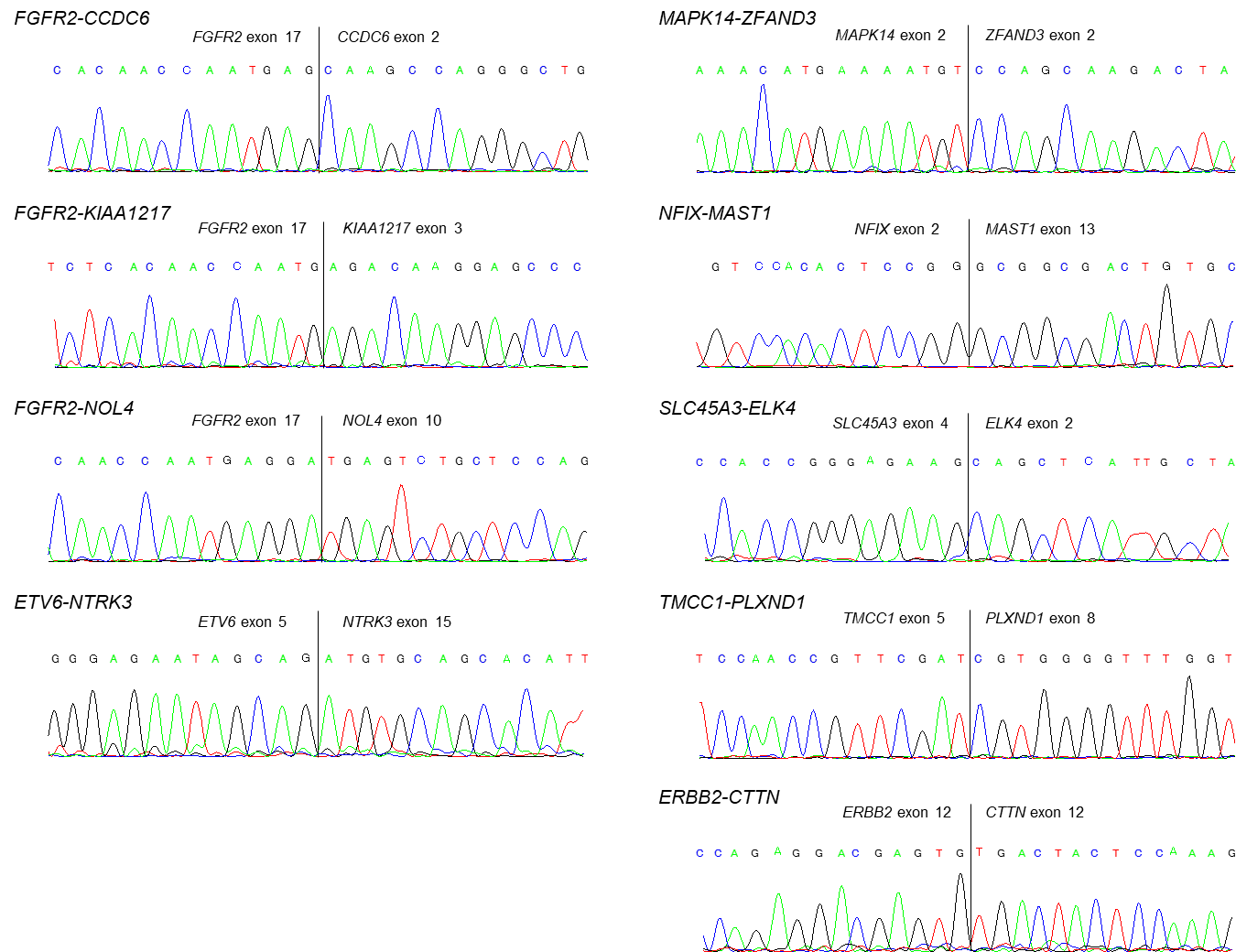
**

**Supplementary Figure 2. Fusion gene confirmation by Sanger sequencing**

The RNA extracted from samples was subjected to reverse transcription PCR, with the respective fusion primer sets to detect fusion transcripts. The electrophoretogram obtained by Sanger sequencing supports the junction sequence of *FGFR2-CCDC6*, *FGFR2-KIAA1217*, *FGFR2-NOL4*, *ETV6-NTRK3*, *MAPK14-ZFAND3*, *NFIX-MAST1*, *SLC45A3-ELK4*, *TMCC1-PLXND1*, and *ERBB2-CTTN*.

**
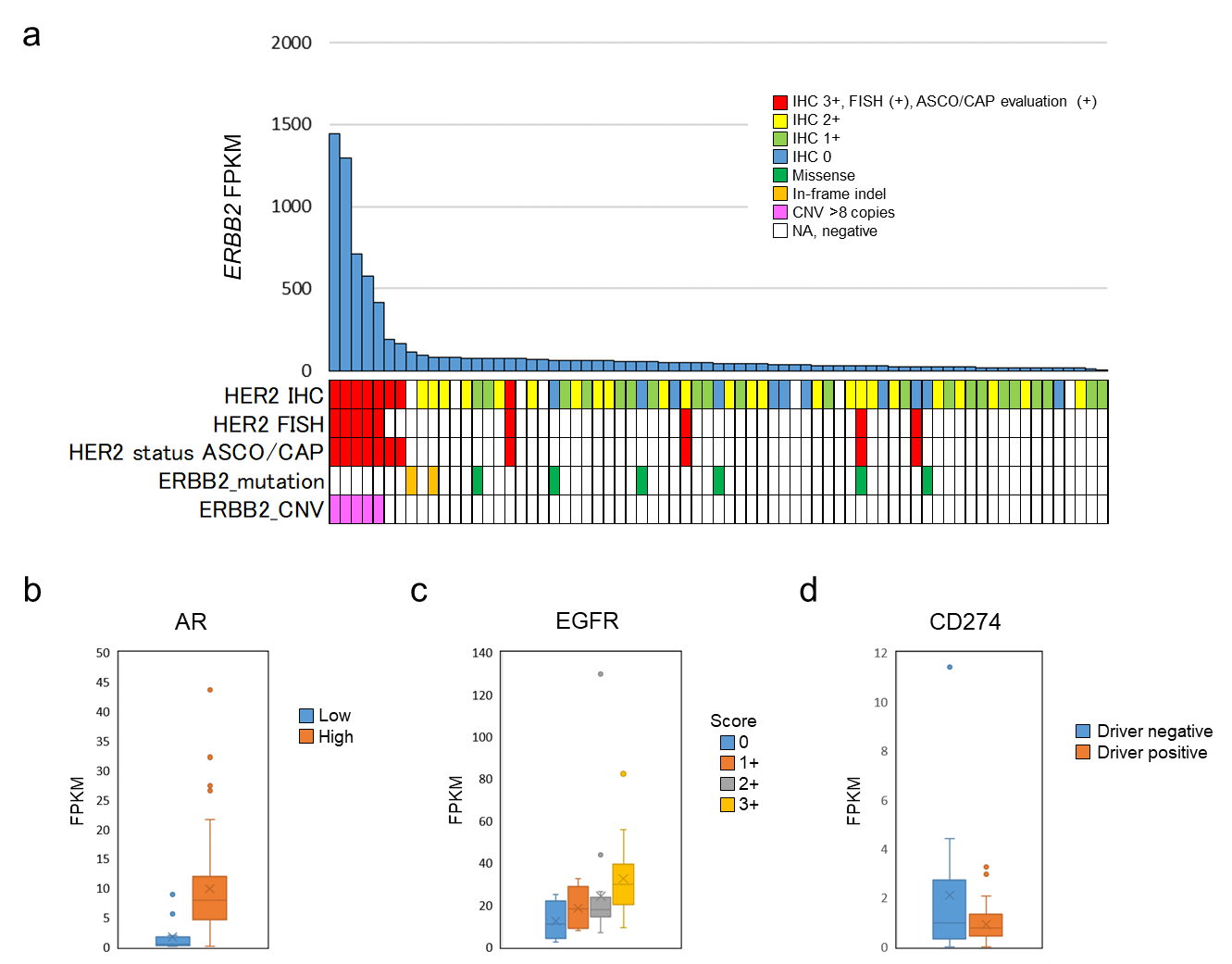
**

**Supplementary Figure 3. Comparison of mRNA expression by RNA-seq with immunohistochemistry**

**(a)** *ERBB2* mRNA expression evaluated by RNA-seq was compared with HER2 IHC and copy number analysis. *ERBB2* copy number was evaluated by FISH and TOP panel. **(b)** **(c)** mRNA expression of *AR* and EGFR evaluated by RNA-seq was compared with the protein expression evaluated by immunohistochemistry. **(d)** mRNA expression of *CD274* was compared between driver-positive and -negative tumors. The elements of boxplots are defined as follows: center line, median; lower bound of box, lower quartile; upper bound of box, upper quartile; lower whiskers, minimum value; upper whisker, maximum value.

**
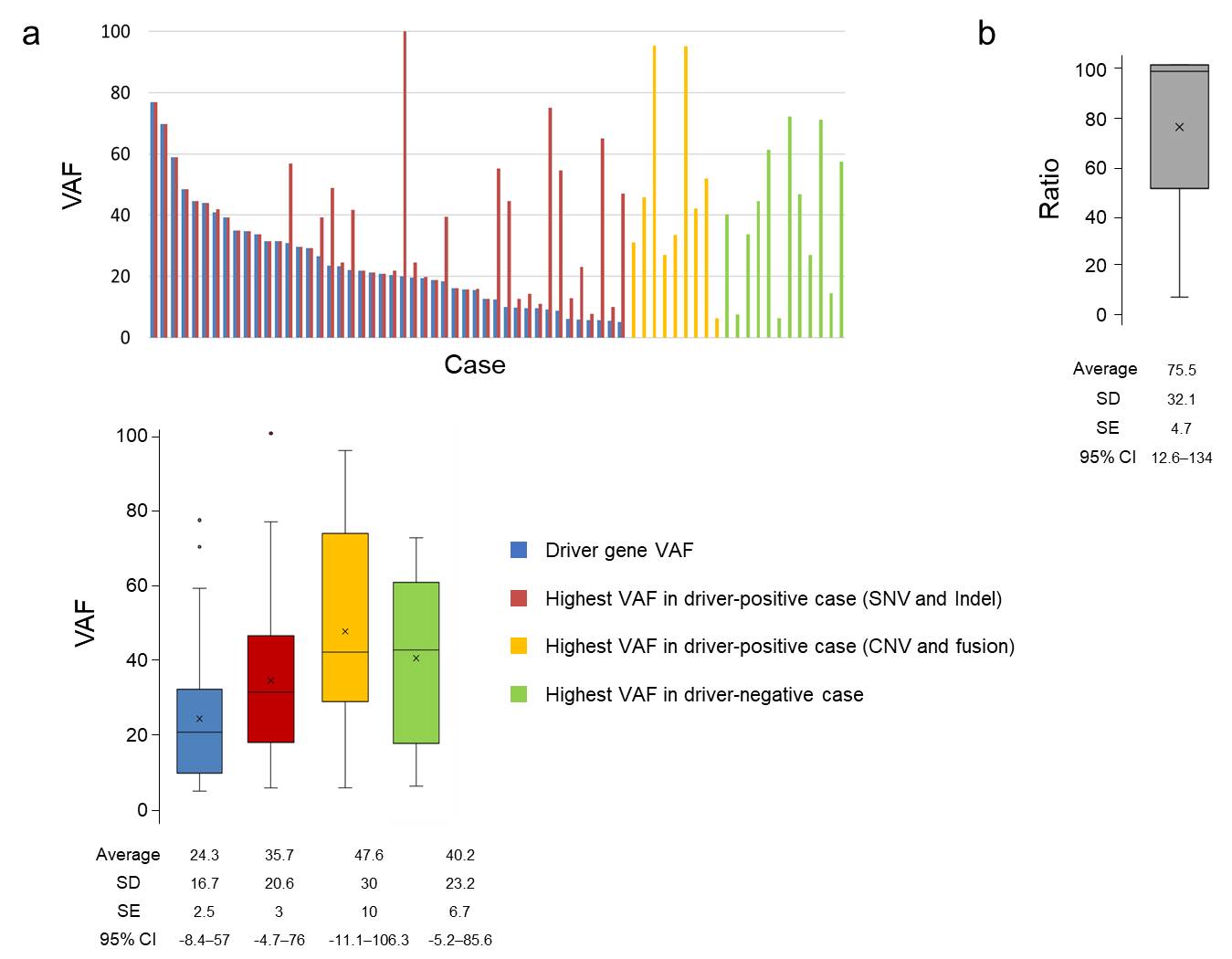
**

**Supplementary Figure 4. VAF comparison in driver-positive and -negative cases**

**(a)** Comparison of variant allele frequency (VAF) of driver mutations with that of the highest VAF observed in each case (upper panel). Box plot of driver gene VAF and highest VAF in driver-positive or -negative cases (lower panel). **(b)** For the cases positive for driver mutations of SNVs and indels, the average ratio of driver VAF to the highest VAF was 75.5 (95% CI = 12.6–134). SD, standard deviation; SE, standard error; CI, confidence interval. The elements of boxplots are defined as follows: center line, median; lower bound of box, lower quartile; upper bound of box, upper quartile; lower whiskers, minimum value; upper whisker, maximum value.

**
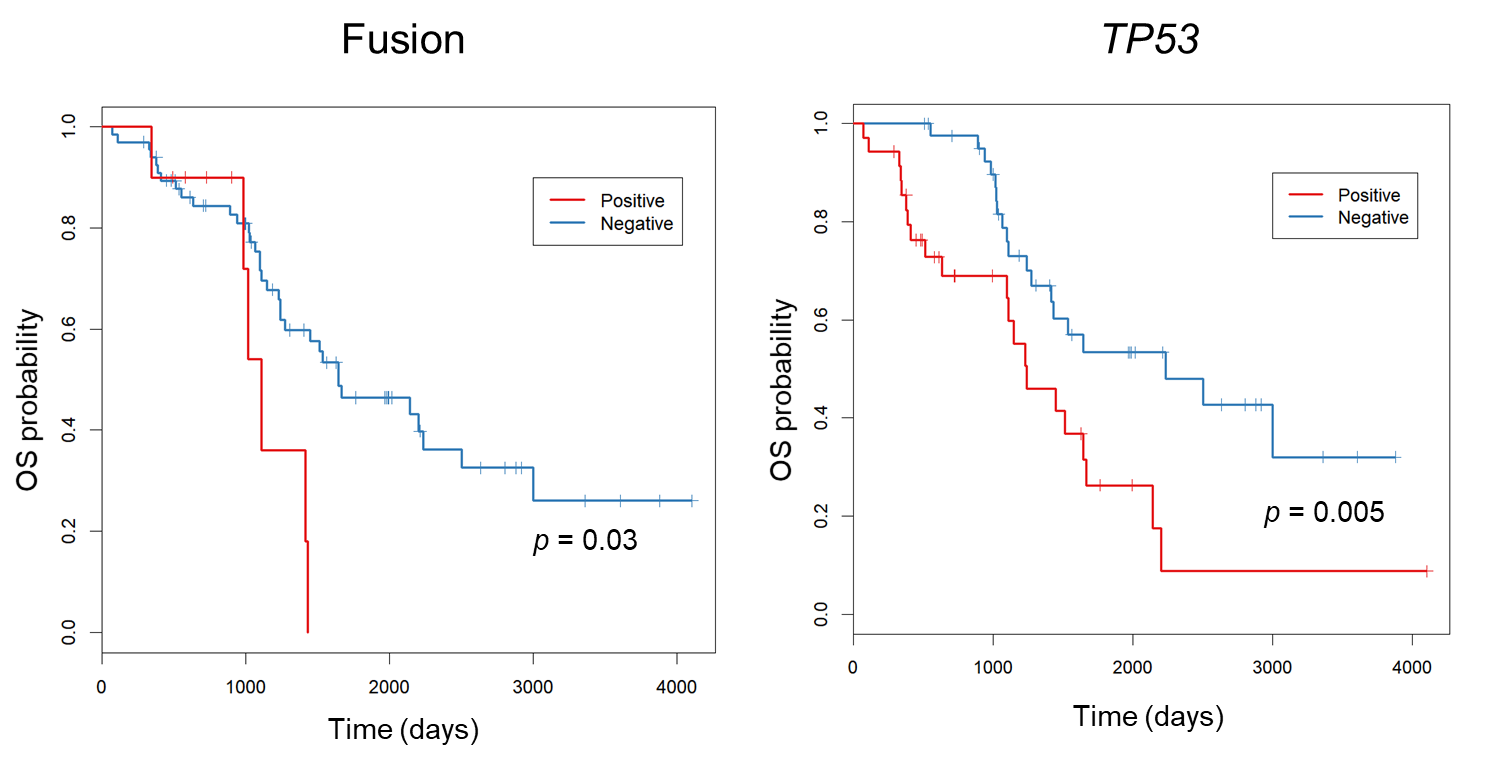
**

**Supplementary Figure 5. Survival comparison by mutation status**

Kaplan–Meier curves of overall survival (OS) in the cohort stratified by the presence of fusion genes or *TP53* mutations.

**
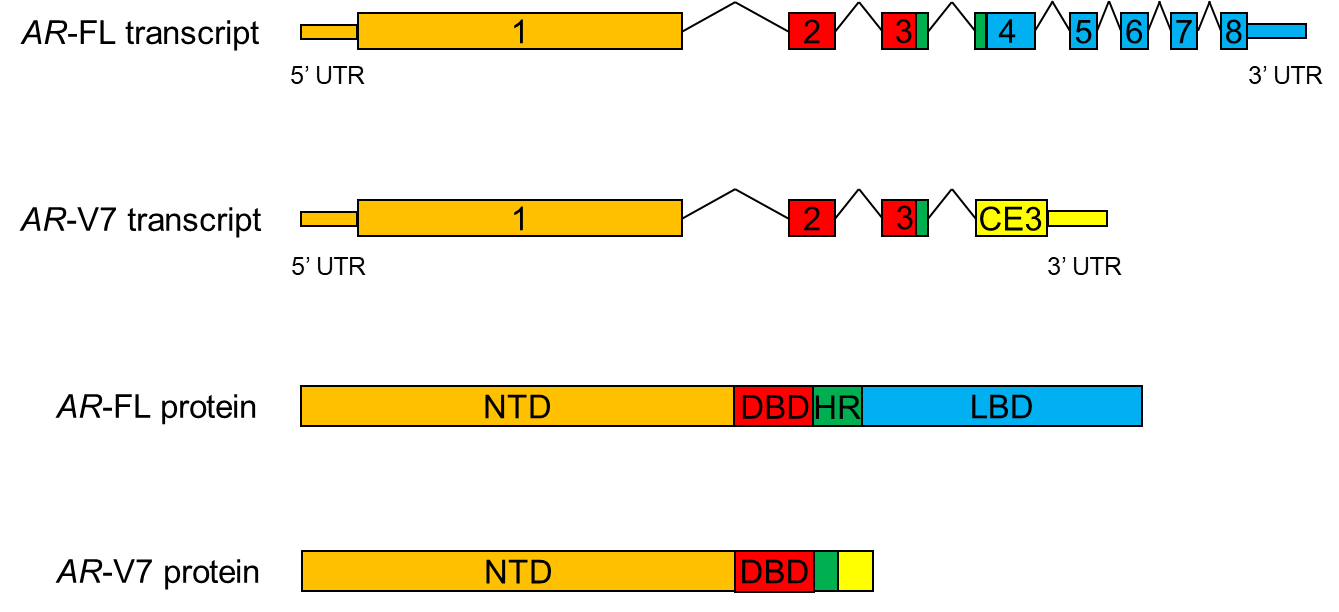
**

**Supplementary Figure 6. Structural differences between full-length AR and AR-V7**

Transcript structures for full-length AR (AR-FL) and splice variant AR-V7. Key structural differences between the AR-FL and AR-V7 are illustrated. The human AR gene has eight canonical exons, color-coded with respect to their AR protein domains: N-terminal domain (NTD) (orange), DNA-binding domain (DBD) (red), hinge region (HR) (green), and ligand-binding domain (LBD) (blue). AR-V7 mRNA retains the first three canonical exons followed by variant-specific cryptic exon 3 (CE3) within intron 3. CE3 splicing results in an LBD-truncated AR-V7 protein because of premature translation termination after 16 variant-specific amino acids. These structural differences between the AR-FL and AR-V7 enable specific detection of AR-FL and AR-V7 by RNA-seq.


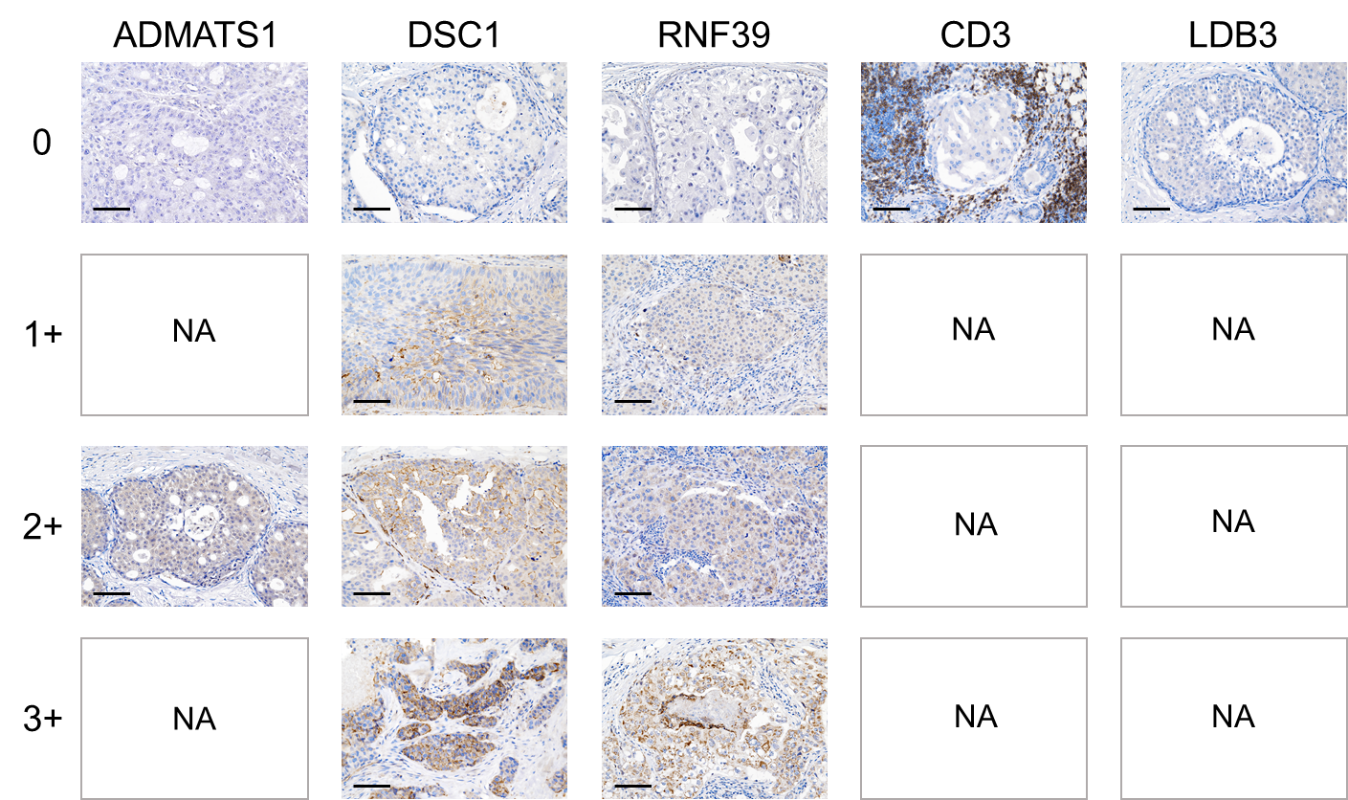


**Supplementary Figure 7. Immunohistochemistry of prognostic markers.**

Immunohistochemistry of ADAMTS1, DSC1, RNF39, CD3 and LDB3 was performed to evaluate protein expression in tumor cells. Staining intensity and ratio of positive cells were assessed. The staining intensity was scored from 0 to 3+ as follows: 0, negative; 1+, mildly positive; 2+, moderately positive; 3+, strongly positive. The magnification of the pictures is ×200. The scale bars represent 100 μm.
